# Primary Fallopian Tube Clear Cell Adenocarcinoma in Pregnancy: Case Presentation and Review of the Literature

**DOI:** 10.1155/2015/183243

**Published:** 2015-05-21

**Authors:** Mohammed Malak, Stephanie Klam

**Affiliations:** ^1^Department of Obstetrics and Gynecology, King Abdulaziz University, Jeddah 22254, Saudi Arabia; ^2^McGill University, Montreal, QC, Canada

## Abstract

Primary fallopian tube cancer in pregnancy is rare and is even more so for the clear cell variant. Our case is the third case of primary fallopian tube cancer in pregnancy and the first case of clear cell adenocarcinoma of the fallopian tube in pregnancy. The patient presented with increasing pelvic pain starting in the second trimester. Serial ultrasound evaluations were performed and revealed a rapidly growing complex adnexal mass adjacent to the uterus. Her pregnancy was further complicated by spontaneous preterm labor and she delivered prematurely per vaginam at 31 weeks. She underwent an urgent laparotomy in the immediate postpartum period for acute aggravation of her right pelvic pain and fever. The diagnosis of tubal clear cell adenocarcinoma was subsequently made on histopathology examination.

## 1. Introduction

Primary fallopian tube cancer in non-BRCA carriers is rare and accounts for 0.3% to 1.8% of genital cancers [1]. Typically, women with fallopian tube cancer are diagnosed in their 6th or 7th decade of life. The most common histological type is papillary serous adenocarcinoma and the clear cell is a rare variant.

One to 2% of pregnancies will be complicated by an adnexal mass that is discovered by ultrasound. The great majorities are functional cysts and only 1% to 3% are malignant. Ultrasonography plays a major role in the management of adnexal masses in pregnancy, especially that tumor marker such as CA 125 loses its accuracy during pregnancy. Simple masses less than 5 cm require no additional follow-up whereas complex or larger masses require close monitoring and surgical intervention if malignancy is suspected. Cytoreductive surgery with lymphnode dissection and chemotherapy is the standard treatment for fallopian tube carcinoma.

## 2. The Case

A 42-year-old female spontaneously conceived naturally after 8 years of unexplained infertility. Her past medical history was significant for chronic hepatitis B and gastric bypass surgery. She had no known allergies. Her sister was known to have breast cancer but she was not screened for BRCA gene mutation.

At 11 weeks' gestation, she presented with right-sided abdominal pain, nausea, vomiting, and anorexia. Physical exam showed right pelvic tenderness and her workup including abdominopelvic ultrasound was unremarkable. Two weeks later she had minimal vaginal bleeding and worsening right-sided abdominal pain; repeat ultrasound examination showed an anterior wall fibroid measuring 40 × 38 × 35 mm and a 38 × 31 mm right-sided adnexal mass; a follow-up scan was planned in 2 weeks.

An uncomplicated genetic amniocentesis for advanced maternal age was performed under ultrasound guidance at 15 weeks and a 43 × 38 × 32 mm pedunculated fibroid on the right lateral wall of the uterus was seen. The right adnexal mass increased in size at subsequent ultrasound examinations and at 23 weeks' gestation it measured 49 × 50 mm and was described as a fibroid.

**Figure 1:**
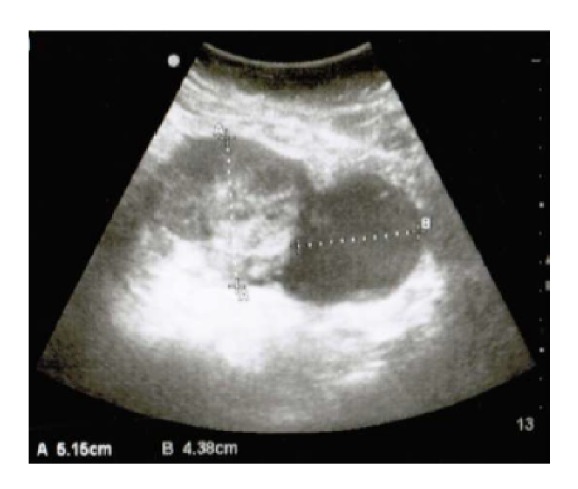
Ultrasonography at 30 weeks showing a complex adnexal mass.

At 30 weeks' gestation she presented to the hospital complaining of severe right-sided abdominal pain. Obstetrical ultrasound examination revealed a single breech fetus, with an estimated fetal weight of 1424 g (19% ile), a mildly reduced amniotic fluid index of 6 cm, and an unusual right adnexal heterogeneous mass with solid (5 cm) and cystic (4 cm) components with good Doppler flow seen adjacent to the uterus where the pain was localized (Figure 1). At this point, and since there was a change in the size and the morphology of the mass, the possibility of an adnexal malignancy was entertained, and further investigations were pursued. The Ca125 was found to be mildly elevated at 89. An MRI was obtained and showed a solid mass measuring 7.4 × 6.3 × 7.0 cm on the right side of the uterus; cranially to the mass, there was a cystic lesion measuring 5.4 × 5.5 × 4.6 cm, which contains several thin-appearing septations. There was no gross nodule within that cystic component. It was unclear whether both lesions were related. It was favored that they are adnexal as there was no clear claw sign from the uterus to the lesions. The patient was discharged on the 4th day of admission with painkillers and follow-up appointment that was arranged for the following week.

Two days after discharge from hospital, at 30 6/7 weeks' gestation, she presented to the birthing centre with spontaneous rupture of membranes in active labor. She was prepared for caesarean delivery; however she delivered spontaneously and precipitously in breech presentation shortly after admission.

Increasing right lower quadrant pain complicated her immediate postpartum course. Ultrasound examination on the first day postpartum showed a 73 × 51 × 75 mm septated right ovarian cystic mass, in addition to a 79 × 63 × 50 mm subserous fibroid medially.

She developed severe pain, fever, and leukocytosis on the second night postpartum and was taken to the operating room after midnight with a working diagnosis of adnexal mass torsion. At midline laparotomy, a large infected mass attached to the lateral pelvic wall, uterus, and adnexa was discovered and removed after peritoneal washings. The appendix was also removed. A frozen section was not obtained since there was no pathologist available at the time of surgery.

The final histopathology diagnosis revealed a right tubal invasive clear cell adenocarcinoma, chronic salpingitis with pseudodecidual reaction, and tuboovarian abscess. She received 3 cycles of neoadjuvant Carbo/Taxol followed by robotic assisted total hysterectomy and left salpingo-oophorectomy, complete right pelvic and para-aortic lymphadectomy, and infracolic omentectomy. Intraoperative findings included a normal cervix, uterus, and left adnexum and no palpable peripheral lymphadenopathy. There were diffuse abdominal adhesions to the right pelvic sidewall, a 1 cm brown nodule at the level of the round ligament, and enlarged lymph nodes that measured up to 2 cm in diameter. Optimal debulking was achieved. The cytology was negative. The histopathology evaluation failed to reveal any residual neoplasia. Postoperatively, she received an additional 3 cycles of adjuvant Carbo/Taxol.

## 3. Comment/Discussion

Primary fallopian tube cancer is the rarest among female genital tract cancers. It accounts for 0.3% to 1.8% of these cancers [1]. Papillary serous adenocarcinoma represents more than 90% of these cancers [2, 3]. Other less common types include clear cell carcinoma, endometrioid cancer, germ cell cancers, and sarcoma [3]. The majority of primary fallopian tube cancers are diagnosed as early stage disease [4, 5]. Preoperative and intraoperative diagnosis are difficult and most patients are initially misdiagnosed, with a final diagnosis being revealed by histopathology [6–8]. The etiology is not well understood but hormonal and possibly genetic factors contribute to the pathophysiology [9]. Approximately 30% of women with fallopian tube cancer have a mutation in BRCA1 or BRCA2 [10].

Diagnostic criteria for the diagnosis of tubal cancer [11] are as follows:

- The main tumor grossly should be in the tube.
- Histologically, the tubal mucosa should be involved with a papillary pattern.
- If the tubal wall is involved to a large extent, transition from benign to malignant tubal epithelium should be identified.

To The best of our knowledge, we are reporting the third primary fallopian tube cancer in pregnancy and the first primary fallopian tube cancer of clear cell adenocarcinoma type in pregnancy.

In 2006, Batra et al. reported primary tubal papillary serous carcinoma in 25-year-old primigravida who attended Wythenshawe Hospital in Manchester. The mass was discovered incidentally at 9 weeks of gestation. The patient became symptomatic 6 weeks later when the mass increased in size. She underwent left salpingo-oophorectomy. The tumor was well differentiated and confined to the tube. Nine weeks postpartum, a second laparotomy was done and included omentectomy and lymphadenectomy with preservation of uterus and right tube and ovary. No adjuvant therapy was introduced and the patient was reported to be fit 2 years after the diagnosis [8].

In 2011, Le et al. reported a primary well differentiated adenocarcinoma of the left tube stage 1A. She was 35-year-old primigravida who presented at term to Shenzhen Nanshan Hospital in China with vaginal bleeding and spontaneous rupture of membrane. She underwent cesarean section during which she underwent left salpingectomy as well. The uterus, left ovary, and right adnexa were looking normal. The author did not mention any ultrasound finding or symptoms relevant to the tubal mass. Shortly postpartum she underwent a debulking surgery followed by chemotherapy [12].

The diagnosis and management of adnexal masses in pregnancy can be challenging. 1% to 2% of pregnancies will be complicated by an adnexal mass that is discovered by ultrasound [13]. The great majority are functional cysts including corpus luteum cyst, follicular cyst, and hemorrhagic cyst. The nonfunctional masses will include benign and malignant pathology (Table 1). Only 1% to 3% of adnexal masses in pregnancy are malignant [14, 15].

In asymptomatic pregnant women, no follow-up is needed for a mass less than 5 cm in diameter, described as a simple cyst or to have benign morphology [16–18]. For cysts discovered in early pregnancy that are larger than 5 cm or have suspicious findings, reimaging is recommended after 16 weeks of gestation. If the lesion decreases in size after 16 weeks, no further management is required. On the other hand, if the lesions are the same or increased in size or changed into complex morphology, reevaluation is recommended and the approach will depend on the ultrasound provisional diagnosis. For example, simple cysts that persist by follow-up ultrasound with no complexity can be managed conservatively only. In cases where masses are designated as persistent complex lesions, MRI is useful.

Unlike other nonobstetrical indications for surgery during pregnancy like gastrointestinal pathologies which can result in major maternal and fetal morbidity and mortality, it is unclear whether conservative management of most persistent adnexal lesions can carry significant risks [19]. For example, in one study they described follow-up of 65,000 women with adnexal masses; only 6 cases required surgery for torsion [19, 20]. Despite these data, surgical intervention during pregnancy is warranted if the radiological features and the growth of the mass are suggestive of malignancy [21, 22]. The recommended time for surgical management during pregnancy is the second trimester, preferably from 16 to 18 weeks. Although this timing is considered ideal, pregnancy loss is a potential complication [21].

Cytoreductive surgery is the treatment of choice for fallopian tube cancer [23]. Generalized pelvic and para-aortic node dissection is recommended over lymph node sampling due to the strong tendency for lymph nodes spread [23–25]. This propensity to lymphatic metastasis makes us think about fallopian tube cancer as a systemic disease similar to ovarian cancer which makes systemic chemotherapy the best option for adjuvant therapy [26]. Barakat et al. reported in 1991 fourfold increase in 5-year survival for patients with advanced-stage disease given cisplatin compared to other case series of patients treated with different agents [27].

## Figures and Tables

**Table 1:** Malignant types of adnexal masses in pregnancy [28].

Histological type	Percentage
Germ cell tumors	45%
Epithelial ovarian tumors	37.5%
Sex cord tumors	10%
Miscellaneous	7.5%

## References

[1] Nordin A. J. (1994). Primary carcinoma of the fallopian tube: a 20-year literature review. *Obstetrical and Gynecological Survey*.

[2] Rosenblatt K. A., Weiss N. S., Schwartz S. M. (1989). Incidence of malignant fallopian tube tumors. *Gynecologic Oncology*.

[3] Terzakis E., Androutsopoulos G., Adonakis G., Zygouris D., Grigoriadis C., Decavalas G. (2011). Fallopian tube primary cancer: report of five cases and review of the literature. *European Journal of Gynaecological Oncology*.

[4] Ajithkumar T. V., Minimole A. L., John M. M., Ashokkumar O. S. (2005). Primary fallopian tube carcinoma. *Obstetrical and Gynecological Survey*.

[5] Baekelandt M., Nesbakken A. J., Kristensen G. B., Tropé C. G., Abeler V. M. (2000). Carcinoma of the fallopian tube: clinicopathologic study of 151 patients treated at The Norwegian Radium Hospital. *Cancer*.

[6] Pfeiffer P., Mogensen H., Amtrup F., Honore E. (1989). Primary carcinoma of the fallopian tube. A retrospective study of patients reported to the Danish Cancer Registry in a five-year period. *Acta Oncologica*.

[7] Rosen A. C., Sevelda P., Klein M. (1994). A comparative analysis of management and prognosis in stage I and II fallopian tube carcinoma and epithelial ovarian cancer. *British Journal of Cancer*.

[8] Batra S., Singh M., Wynn J. S. (2006). An unusual case of primary fallopian tube carcinoma in pregnancy. *International Journal of Gynecological Cancer*.

[9] Pectasides D., Pectasides E., Economopoulos T. (2010). *Fallopian Tube Carcinoma: A Review*.

[10] Vicus D., Finch A., Cass I. (2010). Prevalence of BRCA1 and BRCA2 germ line mutations among women with carcinoma of the fallopian tube. *Gynecologic Oncology*.

[11] Hu C. Y., Taymor M. L., Hertig A. T. (1950). Primary carcinoma of the Fallopian tube. *American Journal of Obstetrics and Gynecology*.

[12] Le A., Shan L., Yuan R., Liu Z., Yang H., Wang Z. (2011). Primary fallopian tube cancer in term pregnancy: a case report. *European Journal of Gynaecological Oncology*.

[13] Chiang G., Levine D. (2004). Imaging of adnexal masses in pregnancy. *Journal of Ultrasound in Medicine*.

[14] Nelson M. J., Cavalieri R., Graham D., Sanders R. C. (1986). Cysts in pregnancy discovered by sonography. *Journal of Clinical Ultrasound*.

[15] Bernhard L. M., Klebba P. K., Gray D. L., Mutch D. G. (1999). Predictors of persistence of adnexal masses in pregnancy. *Obstetrics & Gynecology*.

[16] Koonings P. P., Campbell K., Mishell D. R., Grimes D. A. (1989). Relative frequency of primary ovarian neoplasms: a 10-year review. *Obstetrics and Gynecology*.

[17] Bernhard L. M., Klebba P. K., Gray D. L., Mutch D. G. (1999). Predictors of persistence of adnexal masses in pregnancy. *Obstetrics and Gynecology*.

[18] Agarwal N., Kriplani A., Bhatla N., Gupta A. (2003). Management and outcome of pregnancies complicated with adnexal masses. *Archives of Gynecology and Obstetrics*.

[19] Kilpatrick C. C., Monga M. (2007). Approach to the acute abdomen in pregnancy. *Obstetrics & Gynecology Clinics of North America*.

[20] Yazbek J., Salim R., Woelfer B., Aslam N., Lee C. T., Jurkovic D. (2006). The value of ultrasound visualization of the ovaries during the routine 11–14 weeks nuchal translucency scan. *European Journal of Obstetrics & Gynecology and Reproductive Biology*.

[21] Sayin N. C., Inal H. A., Varol F. G. (2008). Pregnancies complicated by adnexal masses: a case series. *Archives of Gynecology and Obstetrics*.

[22] Swensen R. E., Gov B. A., Koh W.-J., Hoskins W. J., Perez C. A., Young R. C. (2005). Cancer in the pregnant patient. *Principles of Gynecologic Oncology*.

[23] Pectasides D., Pectasides E., Economopoulos T. (2006). Fallopian tube carcinoma: a review. *Oncologist*.

[24] Gadducci A., Landoni F., Sartori E. (2001). Analysis of treatment failures and survival of patients with fallopian tube carcinoma: a cooperation task force (CTF) study. *Gynecologic Oncology*.

[25] Klein M., Rosen A. C., Lahousen M., Graf A. H., Rainer A. (1999). Lymphadenectomy in primary carcinoma of the Fallopian tube. *Cancer Letters*.

[26] Klein M., Rosen A., Lahousen M., Graf A.-H., Rainer A. (2000). The relevance of adjuvant therapy in primary carcinoma of the fallopian tube, stages I and II: irradiation vs. chemotherapy. *International Journal of Radiation Oncology Biology Physics*.

[27] Barakat R. R., Rubin S. C., Saigo P. E. (1991). Cisplatin-based combination chemotherapy in carcinoma of the fallopian tube. *Gynecologic Oncology*.

[28] Glanc P., Brofman N., Salem S., Kornecki A., Abrams J., Farine D. (2007). The prevalence of incidental simple ovarian cysts >or= 3 cm detected by transvaginal sonography in early pregnancy. *Journal of Obstetrics and Gynaecology Canada*.

